# Jacob Bell, Esq.

**Published:** 1859-09

**Authors:** 


JACOB BELL, Esq., expired of laryngeal phthisis, on the 8th of June, at Tunbridge Wells, in the forty-ninth year of his age. At the time of his death he was the President of the Pharmaceutical Society of London, and editor of the Pharmaceutical Journal, both of which owe, in a great measure, their rise and progress to his exertions. His loss will be deeply felt by the chemists and pharmaceutists of England.

